# The epidemiology of do-not-resuscitate orders in patients with trauma: a community level one trauma center observational experience

**DOI:** 10.1186/s13049-015-0094-2

**Published:** 2015-02-03

**Authors:** Kristin Salottolo, Patrick J Offner, Alessandro Orlando, Denetta S Slone, Charles W Mains, Matthew Carrick, David Bar-Or

**Affiliations:** Trauma Research Department, Swedish Medical Center, Englewood, CO 80113 USA; Trauma Research Department, St. Anthony Hospital, Lakewood, CO 80228 USA; Trauma Services Department, St. Anthony Hospital, Lakewood, CO 80228 USA; Trauma Services Department, Swedish Medical Center, Englewood, CO 80113 USA; Rocky Vista University, Aurora, CO 80011 USA; Trauma Services Department, Medical Center of Plano, Plano, TX 75075 USA

**Keywords:** Do-Not-Resuscitate, Trauma, Epidemiology, Outcomes, Elderly

## Abstract

**Background:**

Do-Not-Resuscitate (DNR) orders in patients with traumatic injury are insufficiently described. The objective is to describe the epidemiology and outcomes of DNR orders in trauma patients.

**Methods:**

We included all adults with trauma to a community Level I Trauma Center over 6 years (2008–2013). We used chi-square, Wilcoxon rank-sum, and multivariate stepwise logistic regression tests to characterize DNR (established in-house vs. pre-existing), describe predictors of establishing an in-house DNR, timing of an in-house DNR (early [within 1 day] vs late), and outcomes (death, ICU stay, major complications).

**Results:**

Included were 10,053 patients with trauma, of which 1523 had a DNR order in place (15%); 715 (7%) had a pre-existing DNR and 808 (8%) had a DNR established in-house. Increases were observed over time in both the proportions of patients with DNRs established in-house (p = 0.008) and age ≥65 (p < 0.001). Over 90% of patients with an in-house DNR were ≥65 years. The following covariates were independently associated with establishing a DNR in-house: age ≥65, severe neurologic deficit (GCS 3–8), fall mechanism of injury, ED tachycardia, female gender, and comorbidities (p < 0.05 for all). Age ≥65, female gender, non-surgical service admission and transfers-in were associated with a DNR established early (p < 0.05 for all). As expected, mortality was greater in patients with DNR than those without (22% vs. 1%), as was the development of a major complication (8% vs. 5%), while ICU admission was similar (19% vs. 17%). Poor outcomes were greatest in patients with DNR orders executed later in the hospital stay.

**Conclusions:**

Our analysis of a broad cohort of patients with traumatic injury establishes the relationship between DNR and patient characteristics and outcomes. At 15%, DNR orders are prevalent in our general trauma population, particularly in patients ≥65 years, and are placed early after arrival. Established prognostic factors, including age and physiologic severity, were determinants for in-house DNR orders. These data may improve physician predictions of outcomes with DNR and help inform patient preferences, particularly in an environment with increasing use of DNR and increasing age of patients with trauma.

**Electronic supplementary material:**

The online version of this article (doi:10.1186/s13049-015-0094-2) contains supplementary material, which is available to authorized users.

## Background

The presence of Do-Not-Resuscitate (DNR) orders are increasing over time [1-3] due to advancements in life saving technology and the passage of legislative acts [4] designed to protect patients’ decisions regarding end of life care. The presence and timing of a DNR is associated with disease severity [5] and may be a marker for anticipated poor prognosis [6] or low probability of survival [7,8]. The presence of a DNR is also associated with less aggressive care [9,10] with room for misinterpretation [11,12].

Do-Not-Resuscitate orders in the trauma setting have not been well characterized. The majority of studies describing DNR in a hospital setting have been performed in surgical patients [13-16], intensive care unit (ICU) populations [7,17-19], hemorrhagic stroke patients [20-23], and Medicare populations [5,24-26]. Meanwhile, studies examining DNR in a trauma setting have been limited to subpopulations, including traumatic brain injury (TBI) [27,28], patients admitted to the ICU [29], and severely injured patients requiring immediate transfusion [30]. These previous studies in patients with trauma reported high mortality with DNR (42-99% [29-31]), greater than that observed in general surgical (23-37% [14,15]), stroke (40-64% [10,21,23]), and ICU (51-83% [17-19]) populations. Patients with trauma have also been found to have a lower incidence of DNR established at 5-7%, compared to general surgical (4-65% [13,15]), stroke (22-41% [10,20,23]), and ICU (9-13% [7,17,18]) populations. Identifying characteristics early that may lead to a DNR is increasingly important as DNR orders are becoming more utilized. Due to the paucity of studies of DNR in a trauma setting, we propose to: examine changes in DNR over time, characterize the general population of patients admitted with traumatic injury by the presence and timing of an in-house DNR, examine outcomes by DNR status, and identify predictors of a newly established DNR and DNR established early after injury.

## Methods

### Setting and population

We conducted a retrospective cohort study of all adults (age ≥18) presenting to our Level I Trauma Center between January 1, 2008 and December 31, 2013 in the Denver, Colorado metropolitan area with a traumatic injury (ICD9 diagnostic injury code of 800 – 959.9). Data were entered into the trauma registry (TraumaBase® database, Evergreen Colorado) by dedicated trauma registrars and abstracted electronically. Colorado State criteria were used for inclusion in the trauma registry, as follows: traumatic injury based on ICD-9-CM diagnosis (above), who a) are admitted; b) have an emergency department (ED) disposition of ‘observation’ with an injury severity score (ISS) ≥9 or hospital length of stay ≥12 h; c) are transferred into or out of an acute care facility; d) die; or e) are admitted for missed diagnoses, complications, failed management or iatrogenic injuries identified after a previous hospital encounter [32]. This study was approved by the facility’s Institutional Review Board.

### Outcomes and covariates

The primary exposure variable was the presence of a DNR order, which was recorded in the registry when a signed order sheet was in the patient chart at any point during the hospital stay. A DNR was defined as any order that a) in the event of cardiac arrest, limited the use of chest compressions, cardiodefibrillation, or vasopressor/inotropic support, or b) in the event of respiratory failure, limited the use of intubation and non-invasive mechanical ventilation. At our institution and throughout this manuscript, DNR implies do-not-intubate. We further defined DNR as a pre-existing DNR (recorded as a comorbidity in the trauma registry, pertaining to DNR advance directive) or a DNR established after hospital admission (in-house DNR, recorded as a procedure in the trauma registry). The timing of an in-house DNR was calculated as the number of days from arrival, and stratified as early (within one day of arrival) or late DNR. There is considerable variability among clinicians in discussing DNR, but in general younger patients with low morbidity and no comorbidities are not usually approached.

We examined the following variables, defined as follows: age (18–64 vs. ≥65), gender, Charlson Comorbidity Index (CCI [33], continuous), admission service (surgical service [e.g. trauma service and surgical subspecialties] vs. non-surgical service [e.g. trauma medical service staffed by non-surgical hospitalists]), transferred-in (yes/no), cause of injury (fall vs. all other causes), activation status (trauma activation or alert vs. non-activated), ISS (<16 vs. ≥16), a major injury defined by the abbreviated injury scale score ≥3 using the major AIS regions, ED Glasgow Coma Scale (GCS, 3–8 vs. 9–15) and ED systolic blood pressure (<90 mmHg [hypotension] vs. ≥90 mm Hg), heart rate (<120 beats/min vs. ≥120 beats/min [tachycardia]), and respiratory rate (RR, <12 or >20 breaths/min [abnormal RR] vs. 12–20 breaths/min). Outcomes were examined, including: death, defined as in-hospital mortality or discharge to hospice (end-of-life care); admission to the ICU; a major complication, defined as the presence of any of the following: abdominal compartment syndrome, acute respiratory distress syndrome, acute respiratory failure, coma, cardiac arrest, intubation >48 hours, myocardial infarction, organ failure, surgical infection, pulmonary embolism, pneumonia, sepsis, and stroke [15].

### Statistical analysis

Statistical analyses were performed using SAS® software, version 9.3 (SAS Institute, Cary, NC). Frequencies and descriptive statistics were used to characterize the study population, and presented as percentage (number of observations) [% (n)] or median (interquartile range [IQR]). Chi-square and Wilcoxon rank-sum tests were used to examine differences in covariates. Chi-square test for trend was used to examine changes in DNR and age across arrival year. Multivariate, stepwise logistic regression models were used to identify predictors of establishing an in-house DNR and timing of DNR placement (early vs. late). Entry criteria of p <0.20 and exit criteria of p >0.05 were used for adjustment in the models; all covariates except for AIS and ISS were considered for the models, as AIS and ISS is calculated post-discharge and is used by researchers and not directly by clinicians. Data are presented as odds ratios (OR) and 95% confidence intervals (CI). Because the majority of patients with DNR were ≥65 years, we also stratified the logistic regression analyses by age <65 vs. ≥65. The prevalence of DNR increased by age quartile as follows: 1% (age 18–43), 3% (age 44–64), 16% (age 65–82), 40% (age ≥83); we used the median age of 65 for stratification and ease of interpretability of results rather than expanding or collapsing groups. We explored presence of multicollinearity in the models, defined as a tolerance value less than 0.1 or variance inflation factor greater than 10; no significant multicollinearity was detected. Statistical significance was set at p <0.05 for all analyses. Missing data were not imputed.

## Results

### Overall population

There were 10,053 trauma patients included across the six-year study period. Approximately half of patients were ≥65 years (51%), which increased significantly over the study period (p <0.001, range: 44% to 54%). The majority of patients suffered a fall (60%); followed by motor vehicle accident (15%), bicycle injury (4%) and assault (4%). Severe injuries, based on AIS ≥3, most commonly occurred in limbs (25%, largely due to hip fractures), head (15%), and chest (11%). Fifty-one percent of patients were admitted to a surgical (trauma-41%, orthopedics-8%, neurosurgery-1%, oral maxillofacial-1%, OBGYN-0.5%), the trauma medical (11%), or medical/other (38%) services.

### Do-Not-Resuscitate population

**Figure 1:**
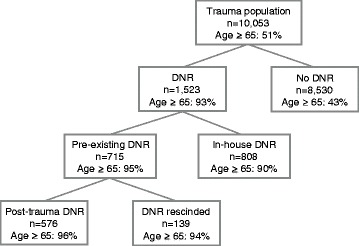
**Distribution of trauma population by Do-not-Resuscitate (DNR) status.**

**Figure 2:**
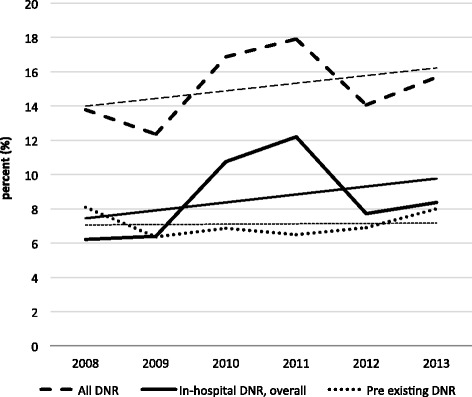
**Changes in Do-not-resuscitate (DNR) over time; trend lines are shown as straight lines using the same dash/weight type as each group presented.**

There were 1,523 (15%) patients with a DNR noted, of which 715 (7%) had a pre-existing DNR and 808 (8%) had a DNR established in-house (Figure 1). DNRs significantly increased over the study period (p = 0.04); however, only DNRs established in-house increased significantly (p = 0.008), while pre-existing DNRs did not (p = 0.31), Figure 2.

Both the in-house and pre-existing DNR populations were predominantly ≥65 years (93%), females (69%) with a fall cause of injury (89%), yet these populations were significantly different for every covariate (Table 1). Patients with a DNR established in-house were more severely injured than patients with a pre-existing DNR based on ISS, presence of severe injuries, trauma activation, surgical service admission, and ED vital signs of GCS, tachycardia, and hypotension (p < 0.05 for all).

**Table 1:** **Patient characteristics by Do-Not-Resuscitate (DNR) status**

**Characteristic, % (n)**	**No DNR (n = 8530)**	**Pre-existing DNR (n = 715)**	**In-house DNR (n = 808)**	**Pre-existing vs. in-house DNR**	**No DNR vs. in-house DNR**
Age ≥ 65	43.02 (3670)	95.24 (681)	90.47 (731)	**< .001**	**< .001**
Female gender	46.07 (3930)	75.24 (538)	63.12 (510)	**< .001**	**< .001**
CCI, median (IQR)	0 (0–1)	1 (0–2)	1 (0–1)	**0.01**	**< .001**
Surgical service admission	55.23 (4711)	15.94 (114)	35.64 (288)	**< .001**	**< .001**
Transferred-in	34.02 (2902)	13.71 (98)	24.38 (197)	**< .001**	**< .001**
Trauma activation	23.90 (2039)	2.80 (20)	13.74 (111)	**< .001**	**< .001**
Fall cause of injury	55.28 (4715)	92.17 (659)	86.01 (695)	**< .001**	**< .001**
GCS 3-8	4.48 (346)	2.40 (14)	12.76 (92)	**< .001**	**< .001**
ISS ≥ 16	17.68 (1505)	11.90 (85)	26.49 (214)	**< .001**	**< .001**
RR <12 or >20 breaths/min	11.06 (774)	9.81 (56)	13.40 (89)	0.05	0.07
Tachycardia^1^	3.26 (229)	1.58 (9)	5.37 (36)	**< .001**	**.004**
Hypotension^2^	2.82 (238)	1.69 (12)	3.63 (29)	**0.02**	0.19
Head injury^3^	14.16 (1208)	12.59 (90)	24.26 (196)	**< .001**	**< .001**
Neck injury^3^	6.26 (534)	3.92 (28)	8.04 (65)	**< .001**	**0.05**
Chest injury^3^	11.52 (983)	4.62 (33)	9.28 (75)	**< .001**	0.05
Abdomen/pelvic injury^3^	3.09 (264)	0.28 (2)	2.10 (17)	**< .001**	0.12
Limb injury^3^	22.70 (1937)	40.14 (287)	32.80 (265)	**0.003**	**< .001**

Significant *p* values (< 0.05) are bolded.

DNR, do not resuscitate; CCI, Charlson comorbidity index; IQR, interquartile range; GCS, Glasgow coma score; ISS, injury severity score; RR, respiratory rate.

^1^Heart rate >120 beats/min.

^2^Systolic blood pressure < 90mmHg.

^3^Abbreviated injury scale score ≥ 3.

There were also significant differences between patients with a DNR established in-house and patients who did not have a DNR (Table 1). Compared to patients without a DNR, the in-house DNR population was older, female, suffered a fall, and was more severely injured based on ISS, GCS, presence of severe head, neck, and limb injury, and tachycardia, yet the in-house DNR population was less frequently activated or admitted to a surgical service (p < 0.001).

Of the 715 patients with a pre-existing DNR, 19% rescinded the DNR post-injury (Figure 1); as such, in the event of cardiac arrest or respiratory failure the clinician would not limit life-sustaining therapy. There were no differences in any covariates for patients with a pre-existing DNR who continued vs. those who rescinded the DNR, except for ED tachycardia (1% vs. 4%, p = 0.04).

Of the 808 patients who had a DNR established in-house, 91% were ≥ 65 years old. The median time to establish an in-house DNR was 0 days; the majority of patients ≥65 years established a DNR within a day of arrival (median: 0, mean: 2), whereas younger patients established a DNR later in the hospital stay (median: 2, mean: 8).

### Predictors of establishing a DNR in-house

The following variables were independently associated with establishing a DNR in-house: age ≥ 65, GCS 3–8, fall injury, ED tachycardia, female gender, and high CCI (Table 2, p < 0.05 for all). A severe neurologic deficit (GCS 3–8) was the most significant predictor of establishing a DNR in-house, with a nearly 13-fold increased odds compared to patients with GCS 9–15.Age was the second most significant predictor, with over 10-fold increased odds of establishing an in-house DNR for patients ≥65 years compared to younger patients, after adjustment.

**Table 2:** **Predictors of a Do-Not-Resuscitate (DNR) established in house (vs. no DNR)**

**Predictor**	**In-house DNR OR (95% CI)**	***p*** **value**
**Overall (n = 9,338)**		
GCS 3-8	12.64 (8.66 – 18.45)	**< 0.001**
Age ≥ 65	10.44 (7.52 – 14.50)	**< 0.001**
ED tachycardia^1^	2.90 (1.85 - 4.53)	**< 0.001**
Fall cause of injury	2.87 (2.06 - 4.00)	**< 0.001**
Female gender	1.28 (1.05 - 1.55)	**0.02**
CCI	1.19 (1.12 - 1.27)	**< 0.001**
**Age ≥ 65 (n = 4401)**		
GCS 3-8	4.43 (3.92 – 10.55)	**< 0.001**
Fall cause of injury	2.81 (1.86 - 4.24)	**< 0.001**
ED tachycardia^1^	2.49 (1.40 - 4.42)	**0.002**
Female gender	1.26 (1.02 - 1.55)	**0.03**
CCI	1.18 (1.10 - 1.26)	**< 0.001**
**Age 18–64 (n = 4937)**		
GCS 3-8	29.84 (16.52 – 53.92)	**< 0.001**
Fall cause of injury	2.80 (1.58 – 4.94)	**< 0.001**
ED tachycardia^1^	3.13 (1.54 – 6.38)	**0.002**
CCI	1.35 (1.14 - 1.64)	**< 0.001**

Entry criteria of p < 0.20 and exit criteria of p > 0.05.

OR, odds ratio; CI, confidence interval; GCS, Glasgow coma score; ED, emergency Department; CCI, Charlson comorbidity index.

^1^Heart rate > 120 beats/min.

The same variables that predicted the presence of an in-house DNR in our general population were observed in the ≥65 subset (Table 2); likewise, those same predictors were observed in the younger subset, except female gender (Table 2).

### Predictors of early timing of DNR in-house

The following variables were independently associated with early placement of a DNR in-house: age ≥ 65, female gender, non-surgical service admission, and transfer-in (Table 3). Surprisingly, severity of injury was not associated with early placement of a DNR, including GCS, ED vital signs and activation status. In the ≥65 years subset, female gender, non-surgical service admission, and transfer were independently associated with an early DNR (Table 3). Only admission to a non-surgical service increased the odds of an early DNR in the subset of patients < 65 years.

**Table 3:** **Predictors of an early (within 1 day) in-house Do-Not-Resuscitate (DNR) vs. late DNR**

**Predictor**	**Early DNR OR (95% CI)**	***p*** **value**
**Overall (n = 808)**		
Age ≥ 65	2.35 (1.25 - 4.41)	**0.01**
Female gender	2.32 (1.52 - 3.52)	**< 0.001**
Non-surgical service	2.44 (1.52 – 3.93)	**< 0.001**
Transferred in	1.75 (1.02 – 3.00)	**0.04**
**Age ≥ 65 (n = 731)**		
Female gender	2.54 (1.62 – 3.99)	**< 0.001**
Non-surgical service	2.37 (1.43 - 3.94)	**< 0.001**
Transferred in	1.99 (1.07 - 3.72)	**0.03**
**Age < 65 (n = 77)**		
Non-surgical service	5.25 (1.24 – 22.24)	**0.02**

Entry criteria of p < 0.20 and exit criteria of p > 0.05.

DNR, do-not-resuscitate; OR, odds ratio; CI, confidence interval.

### DNR and outcomes

There were 455 deaths (5%), including 118 patients who were discharged to hospice. Five percent of patients developed a major complication, the most common was pneumonia (15%), acute respiratory failure (9%) and pulmonary embolism (3%). Admission to the ICU was common (17%), with an ICU LOS of 3 (2–6) days.

**Figure 3:**
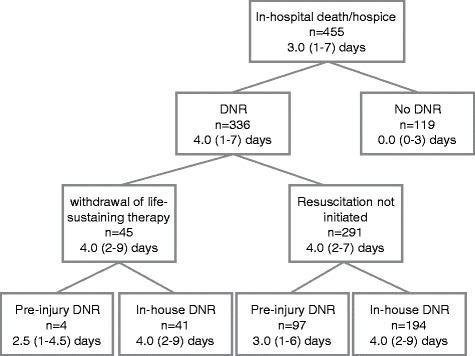
**Distribution of Do-not-Resuscitate (DNR) in patients who died, including median (interquartile range) days from arrival to death.**

Three-fourths of patients who died had a DNR (Figure 3). Median time to death was shortest in patients who did not have a DNR (0 days; presumably because they died before a DNR could be made), followed by patients with a pre-existing DNR and lastly patients who established the DNR in-house. Forty-five (3%) patients with a DNR had withdrawal of life sustaining therapy. Compared to patients with a DNR who did not withdraw life-sustaining therapy, patients who withdrew life sustaining therapy were younger (age < 65: 27% vs. 7%), male (60% vs. 30%), transferred in (42% vs. 19%), suffered a non-fall injury (29% vs. 11%), and were more severely injured (GCS 3–8, 47% vs. 7%; ISS ≥16, 69% vs. 18%; admitted to a surgical service: 80% vs. 26%; trauma alert/activation: 44% vs. 8%; abnormal RR, 40% vs. 11%; tachycardia, 11% vs. 3%, hypotension, 12% vs. 2%; and higher percent with severe head, neck, chest, and abdomen/pelvic injuries, p < 0.01 for all.

Unadjusted outcomes by DNR status are shown in Table 4. As expected, mortality was significantly greater in patients with a DNR, particularly those with a DNR established later during hospitalization. Similarly, patients with a DNR had greater incidence of major complication than patients without a DNR, especially with an in-house and a late DNR. Admission to the ICU was similar in patients with and without a DNR. In patients with a pre-existing DNR, mortality was lower in patients who rescinded the DNR post-injury while development of major complications and ICU admission were similar.

**Table 4:** **Unadjusted outcomes by Do-Not-Resuscitate (DNR) status**

**Predictor**	**N**	**Death/discharge to hospice**	**Major complication**	**ICU admission**
DNR	1523	22.06 (336)	8.29 (106)	18.71 (285)
Pre-existing	715	14.13 (101)	3.09 (18)	5.73 (41)
Continued DNR	576	15.63 (90)	6.08 (35)	3.30 (16)
Rescinded DNR	139	7.91 (11)	4.32 (6)	2.04 (2)
In-house	808	29.08 (235)	12.66 (88)	30.20 (244)
Early (within 1 day)	536	20.71 (111)	4.69 (21)	17.72 (95)
Late (>1 day)	207	52.17 (108)	30.60 (56)	61.35 (127)
No DNR	8530	1.40 (119)	5.02 (361)	17.02 (1452)

## Discussion

Do-Not-Resuscitate orders in patients suffering traumatic injury have not been sufficiently and completely characterized. We analyzed over 10,000 patients with traumatic injury and demonstrated that a DNR was prevalent at 15% and use of in-hospital, post injury DNR orders increased over the course of the study. DNRs established in-house were most associated with advanced age and poor GCS. In-hospital DNRs were established within 1 day in 72% of patients, particularly in those ≥65 years old. Age was the most significant predictor of establishing an in-house DNR within one day of arrival, while none of the expected covariates measuring severity were associated with early DNR, including GCS, ED vital signs, and trauma activation. Interestingly, non-surgical service admission was associated with early DNR; this finding may reflect medicine physician’s training of end-of-life care discussions versus that of surgeons. The variables identified that independently predicted a DNR in-house and an early DNR could be used to direct clinicians on who and when to approach about signing a DNR. These findings might help ethics committees establish the appropriateness of making a patient DNR when this is controversial or the family is uncertain about this choice.

Our study not only included a broad cohort of patients with traumatic injury, but was also able to differentiate pre-existing DNR orders vs. those established in-house, and established the relationship between patient characteristics, timing of DNR, and outcomes. Previous studies did not differentiate whether the DNR was from existing advance directives or whether it was established after sustaining traumatic injury. The increasing age of the trauma population will likely lead to an increase in patients carrying DNR advance directives: National trends report the population ≥65 years has notably increased over time and grew at a faster rate than the total population [34].

This is the most comprehensive description of DNR in a trauma setting. Most studies on DNR in trauma patients have been performed in a non-trauma setting where patients’ end-of-life status are known, and it is more practical to provide pain and symptom management and goals of care based on patients’ wishes. There may be neglect of palliative care in trauma because of the focus on aggressive treatment and resuscitation [35]. Due to the nature of their condition, patients with trauma usually receive aggressive treatments and early resuscitation because there is uncertainty about the ultimate outcome following traumatic injury.

Studies examining DNR following trauma have largely limited the population to severely injured patients based on ICU admission [29], immediate blood transfusion [30], or presence of TBI [27,28]. In our cohort, most patients with a DNR established post-injury did not have a TBI (69%) or go to the ICU (70%); thus, limiting trauma populations to those with severe injuries has resulted in an incomplete picture of DNR following traumatic injury. The prevalence of a DNR was higher in our general trauma population (15%) compared to other severely injured trauma subpopulations (5-7%), likely due to the age of our trauma population or the increasing use of DNR as an order to protect a patient’s autonomy.

DNR is used more frequently in elderly patients independent of disease prognosis in non-trauma settings, including 12% of hospitalized patients ≥65 years [26], 13% of stroke patients ≥50 years [36], and 13% of emergency surgical ICU patients whose mean age was 63 years [19]. Our retrospective study was unable to determine if DNR is overused in elderly patients or underused in younger patients with trauma, but our findings demonstrating that > 90% of in-house DNRs were established in patients ≥65 years reflect two likely explanations: 1- an age bias exists in which clinicians approach older patients, believing they are more likely to want comfort measures rather than aggressive resuscitation, whereas younger patients will sign a DNR only when all other therapeutic options have been exhausted; 2- the older population has contemplated death, resuscitation and DNR and previously discussed their wishes with family or has a DNR advance directive in place.

We found that the presence of a DNR did not appear to be a sign of eventual death, as only 22% of patients with DNR died in our study compared with observed rates of 88% in trauma patients admitted to the ICU [29] and 99% for those requiring blood transfusion [30]. This finding may reflect the increasing use of DNR as an order to protect a patient’s autonomy even in conditions, such as traumatic injury, that view withdrawal of life sustaining therapy as a failure [37].

The primary limitation of our study is that information on the reason for DNR placement was not recorded electronically, nor was information on presence of living wills and power of attorney. As such, we did not know whether the decision to sign the DNR was made by the patient, surrogate, or a proxy. Second, we did not determine the causal relationship between development of a major complication, ICU admission, and placement of a DNR. Third, our trauma center has a low incidence of penetrating injury (3%) and high proportion ≥65 years (51%), thus making our results potentially less generalizable to other trauma centers. This study was also conducted at a single institution, further limiting the generalizability and likely resulting in population bias. Because of these limitations, several questions arise for future research. Primarily, what was the reason for end-of-life decisions, were DNRs established late in the hospital course due to exhaustion of therapeutic options, or were DNRs established early in the hospital course due to physician perceptions of patient prognosis, age bias, or the increasing use of DNR as a legal order used to respect patient wishes?

## Conclusions

At our level I community trauma center, DNR orders are increasing, reflecting the greater age of our population, the increasing use of DNR for quality of life and patient autonomy, or both. A DNR order that was executed in-house, post injury was associated with greater age, severe injury (GCS, tachycardia), injury due to falls, and number of comorbidities in our general trauma population. Age ≥65 was the greatest predictor of establishing a DNR after injury, particularly early in the hospital course. As expected, mortality was greater in patients with DNR than those without, and poor outcomes were greatest in patients with DNR orders executed later in the hospital stay. It is important for physicians and trauma patients to understand the distribution and determinants of DNR orders and their associated outcomes following trauma to inform decisions for limiting life-sustaining therapy.

## Abbreviations

DNR: Do-not-resuscitate
ICU: Intensive care unit
TBI: Traumatic brain injury
ISS: Injury severity score
ED: Emergency department
LOS: Length of stay
CCI: Charlson comorbidity index
RR: Respiratory rate
IQR: Interquartile range
CI: Confidence interval
